# CRISPR/Cas9 targeting of GPRC6A suppresses prostate cancer tumorigenesis in a human xenograft model

**DOI:** 10.1186/s13046-017-0561-x

**Published:** 2017-06-28

**Authors:** Ruisong Ye, Min Pi, John V. Cox, Satoru K. Nishimoto, L. Darryl Quarles

**Affiliations:** 1 Department of Medicine, University of Tennessee Health Science Center, 19 S Manassas St., Memphis, TN 38163 USA; 2 Department of Microbiology, Immunology and Biochemistry, University of Tennessee Health Science Center, 19 S Manassas St., Memphis, TN 38163 USA

**Keywords:** Osteocalcin, GPRC6A, CRISPR/Cas9, Prostate cancer

## Abstract

**Background:**

GPRC6A is implicated in the pathogenesis of prostate cancer, but its role remains uncertain because of a purported tolerant gene variant created by substitution of a K..Y polymorphism in the 3rd intracellular loop (IL) that evolved in the majority of humans and replaces the ancestral RKLP present in 40% of humans of African descent and all other species.

**Methods:**

We determined whether the K..Y polymorphism is present in human-derived prostate cancer cell lines by sequencing the region of the 3rd IL and assessed the cellular localization of a “humanized” mouse GPRC6A containing the K..Y sequence by immunofluorescence. We assessed functions of GPRC6A in PC-3 cells expressing endogenous GPRC6A and in GPRC6A-deficient PC-3 cells created using CRISPR/Cas9 technology. The effect of GPRC6A on basal and ligand stimulated cell proliferation and migration was evaluated in vitro in wild-type and PC-3-deficient cell lines. The effect of editing GPRC6A on prostate cancer growth and progression in vivo was assessed in a Xenograft mouse model implanted with wild-type and PC-3 deficient cells and treated with the GPRC6A ligand osteocalcin.

**Results:**

We found that all of the human prostate cancer cell lines tested endogenously express the “K..Y” polymorphism in the 3rd IL. Comparison of mouse wild-type GPRC6A with a “humanized” mouse GPRC6A construct created by replacing the “RKLP” with the “K..Y” sequence, found that both receptors were predominantly expressed on the cell surface. The transfected “humanized” GPRC6A receptor, however, preferentially activated mTOR compared to ERK signaling in HEK-293 cells. In contrast, in PC-3 cells expressing the endogenous GPRC6A with the “K..Y” polymorphism, the ligand osteocalcin stimulated ERK, AKT and mTOR phosphorylation, promoted cell proliferation and migration, and upregulated genes regulating testosterone biosynthesis. Targeting GPRC6A in PC-3 cells by CRISPR/Cas9 significantly blocked these responses in vitro. In addition, GPRC6A deficient PC-3 xenografts exhibited significantly less growth and were resistant to osteocalcin-induced prostate cancer progression compared to control PC-3 cells expressing GPRC6A.

**Conclusions:**

Human GPRC6A is a functional osteocalcin and testosterone sensing receptor that promotes prostate cancer progression. GPRC6A may contribute to racial disparities in prostate cancer, and is a potential therapeutic target to develop antagonists to treat prostate cancer.

**Electronic supplementary material:**

The online version of this article (doi:10.1186/s13046-017-0561-x) contains supplementary material, which is available to authorized users.

## Background

Prostate cancer is the most commonly diagnosed cancer and the second leading cause of cancer-related deaths in men in the United States [1]. Androgen depravation therapy (ADT) is the initial treatment for this disorder, but the disease often progresses to more clinically aggressive castrate-resistant prostate cancer (CRPC). There is an unmet need to understand the molecular pathogenesis of CRPC and identify novel pharmacological targets to design new treatments for prostate cancer [2].

GPRC6A is a widely expressed G-protein coupled receptor that is a master regulator of energy metabolism, and sexual reproduction [3–13]. Ligands for GPRC6A include osteocalcin (Ocn), a bone derived hormone [15], testosterone (T), basic amino acids, such as L-arginine (L-Arg), and cations, such as calcium and zinc [16]. While testosterone can be converted to DHT to activate androgen receptors, and other sensing mechanisms exist for L-Arg [17], and cations [16], GPRC6A is the only identified receptor for osteocalcin [5]. GPRC6A is expressed in multiple mouse tissues, where it regulates metabolic processes and stimulates the release of hormones, including insulin from β-cells [3, 18], testosterone from Leydig cells [6], glucagon-like peptide-1 (GLP-1) from intestinal cells [7, 8], adiponectin from adipocytes [9, 19], and interleukin 6 (IL-6) from myocytes [10].

There is emerging evidence that GPRC6A is involved in the pathogenesis of prostate cancer. First, GPRC6A is increased in human prostate cancer cell lines and in a subset of human prostate cancer tumors [12]. Second, activation of GPRC6A stimulates cell proliferation and chemotaxis in human prostate cancer cells [12], and promotes prostate cancer epithelial-mesenchymal transition [20], whereas knockdown of GPRC6A inhibits prostate cancer cell migration and invasion [12]. Third, human genome wide association studies (GWAS) link single nucleotide polymorphisms (SNPs) in the GPRC6A locus with the development of prostate cancer in the Japanese, and Chinese population [21–23]. More recently, the SNP rs1606365 GPRC6A was found to be associated with aggressive prostate cancer [20]. Fourth, genetic ablation of GPRC6A attenuates prostate cancer progression in the TRansgenic Adenocarcinoma of the Mouse Prostate (TRAMP) model [12], Fifth, high serum levels of osteocalcin is a biomarker of prostate cancer [24], and the autocrine/paracrine release of osteocalcin may stimulate prostate cancer growth and progression through activation of GPRC6A [25]. Finally, GPRC6A mediates the non-genomic, rapid signaling responses to testosterone [26], leading to activation of PI3K/Akt pathways that are implicated in androgen receptor (AR)-independent progression and therapeutic resistance of prostate cancer [27]. GPRC6A may provide a molecular basis for the association between Metabolic Syndrome and the risk of prostate cancer [14].

A direct role of GPRC6A in the pathogenesis of human prostate cancer, however, remains to be established with certainty. Some observations even suggest that evolutionary divergence of GPRC6A in humans has led to a loss-of-function tolerant gene variant with little pathophysiological effects [28–30]. GPRC6A is not identified in gene expression signatures that predict prostate cancer disease severity in humans [31]. Moreover, questions regarding the functions GPRC6A and its ligands have been raised [28, 29, 32]. Finally, intracellular retention of human GPRC6A has been reported [29, 33], suggesting that the human GPRC6A receptor may have diminished cell surface expression and function.

In the current study, we examined the function of human GPRC6A in prostate cancer progression in vitro and in vivo. We demonstrated that GPRG6A is expressed in human prostate cancer cell lines and has an evolutionarily divergent polymorphism that does not affect cell surface expression but preferentially enhances mTOR signaling. We also used clustered regularly interspaced short palindromic repeats (CRISPR) and CRISPR-associated protein 9 nuclease (Cas9) (CRISPR/Cas9) [34] to disrupt the GPRC6A gene in the human prostate cancer cell line (PC-3). We found that editing the endogenous GPRC6A gene using CRISPR/Cas9 inhibits osteocalcin activation of ERK, AKT, and mTOR signaling pathways, and cell proliferation and migration in vitro. Finally, we found that GPRC6A mediates prostate cancer progression in vivo by assessing the response to osteocalcin in human prostate cancer xenograft models of cells expressing endogenous GPRC6A or with CRISPR/Cas9 mediated deletion of GPRC6A. Taken together, our findings support a role for GPRC6A and osteocalcin in prostate cancer, and define a potential therapeutic target to suppress prostate cancer progression.

## Methods

### Cell culture and reagents

Human prostate cancer cell lines, PC-3, DU145, LNCap and 22Rv1 were cultured in the RMPI1640 medium (Life Technologies, #A10491-01) supplemented with 10% FBS (ATLANTA biologicals; S11150), 10 units/ml penicillin, and 100 μg/ml streptomycin (Life Technologies; 15140).

Decarboxylated osteocalcin was produced and purified as described previously [5].

Human GPRC6A cDNA was RT-PCR-amplified from human kidney total RNA and cloned into the expression vector pcDNA3.1(+)-Puro as previously described [18]. Mouse GPRC6A cDNA construct was a gift from Dr. Hampson [35].

### Immunofluorescence localization studies

HEK-293 cells stably transfected with a myc-tagged version of wild type mouse GPRC6A or the mutant in which KY was substituted for RKLP sequence in the 3rd intracellular loop were fixed by incubating the cells in 4% paraformaldehyde in phosphate buffered saline (PBS) for 10 min at room temperature. The cells were then permeabilized by incubation in PBS containing 0.05% saponin for 10 min. The cells were then washed and incubated with a mouse anti-myc tag antibody (Cell Signalling) followed by donkey anti-mouse IgG conjugated to Alexa 488 (Molecular Probes). After washing the cells were imaged using a Zeiss AxioImager II microscope.

### Isolation of membrane fractionation

The membrane protein was extracted from PC-3 cells using Mem-PER™ Plus Membrane Protein Extraction Kit (ThermoScientific; 89842), following the manufacturer’s instruction. Total protein was extracted using Cell Extraction Buffer (ThermoScientific; FNN0011). 15 μg of total or membrane protein was loaded for Western blot analysis.

### Site-directed mutagenesis

The KY mutation in mGPRC6A was introduced using the QuikChange site-directed mutagenesis kit (Stratagene) with specific oligonucleotides, mGPRC6A.KYfor: 5’-GCATTCAAGGGCAAATATGAGAATTACAACGAAGCC-3’ and mGPRC6A.KYrev: 5’-GGCTTCGTTGTAATTCTCATATTTGCCCTTGAATGC-3’. All final constructs were verified by sequencing on both strands.

### RT-PCR

The primer set used to amplify this specific region of GPRC6A are: hGPRC6A.F2240, 5’-GAATGTCTCCTTGCCCAGAG-3’ and hGPRC6A.R2734, 5’-TCTTTGCTTTTCTGCCAGGT-3’. The RT-PCR products were isolated and purified using QIAquick Gel Extraction kit (Qiagen) and sequenced using the hGPRC6A.F2240 primer.

### Targeting GPRC6A in PC-3 cells using CRISPR/Cas9

The short guide RNA (sgRNA; sgRNA1 and 3) targeting exon 3 of the human GPRC6A gene (NM_001286354.1) were designed by Zhang Lab (http://crispr.mit.edu/), and synthesized to make the lenti-GPRC6A-sgRNA3-Cas9 constructs as described previously [36]. In brief, the 25-bp DNA oligos containing the 20 bp target sequence and *Bsm*bI sticky end were annealed and inserted into the lentiCRISPR-v2 plasmid (Addgene; 52961) digested with *Bsm*bI (Fermentas). The DNA sequence for generating sgRNA1 and sgRNA3 are as follows: sgRNA1 forward: 5’- CACCg*ATCCCGAATGGCATAACCA*-3’ and sgRNA1 reverse: 5’-AAACTGGTTATGCCATTCGGGATc-3’, sgRNA3 forward: 5’-CACCg*CCCCAACGCCTTTCAACCAT*-3’, sgRNA3 reverse: 5’-AAACATGGTTGAAAGGCGTTGGGGc-3’. For the control plasmid, no sgRNA sequence was inserted into the construct. GPRC6A edited cells and control cells were selected using 4 μg/ml puromycin. To determine the genome-editing effect, genomic DNA was extracted from the GPRC6A edited cells and control cells, and analyzed by sequencing PCR amplification products of the edited region. The PCR primers are as follows: hGPRC6A.Fmut: 5’-GCCCAGGTTAATGTCATTGT-3’; and hGPRC6A.Rmut: 5’-GGTAGGTCAGAGTAGGAAGTG-3’. The editing of GPRC6A was validated by western blot and Real-time PCR.

### Cell proliferation and migration assays

For proliferation assays, 4 ×10^3^ GPRC6A edited cells and control cell were seeded in the each well of 96-well plate, with or without various concentrations of osteocalcin (10, 50, 100, and 150 ng/ml), and the MTT dye production method was used to measure for cell proliferation (Cayman Chemical, # 10009365). A cell migration assay was performed using the CytoSelect 24-well cell migration Colorimetric Format assay (Cell Biolabs; CBA-100-C), following the manufacturer’s instructions. In brief, each well contained a Boyden chamber with an 8-μm pore polycarbonate membrane and media containing 10% FBS with or without 100 ng/ml osteocalcin. Chambers were seeded with 10^5^ GPRC6A edited cells or control cells expressing GPRC6A in serum free media and incubated at 37 °C 24 h. Cells within wells were washed away, and migratory cells were stained and counted using a microplate reader at OD 570 nm.

### Western blot

Western blots were performed as previously described [37]. The antibodies used for these analyses were the following: GPRC6A antibody (ASSAY BIOTECHNOLOGY, #G321); Phospho-p44/42MAPK (ERK1/2)(Thr202/Tyr204) antibody (Cell Signaling,#9101); p44/42MAPK (ERK1/2) (Cell Signaling,#9102); Phospho-mTOR (Ser2448) antibody (Cell Signaling,#2971); Phospho-Akt (Ser473) antibody (#9271).

### Real-time PCR

All the primers used for this study are listed in Table 1. Samples were amplified for 40 cycles in an iCycler iQ real-time PCR detection system (Bio-Rad) with an initial melt at 95^o^ C for 10 min, followed by 40 cycles of 95^o^ C for 15 s and Tm for 1 min. PCR product accumulation was monitored at multiple points during each cycle by measuring the increase in fluorescence caused by the binding of SYBR Green I to double-stranded DNA. The threshold cycle [38] of tested-gene product from each group was normalized to the Ct of cyclophilin A, the 2^-ΔΔCt^ method was employed to quantify and normalize the expression data.

**Table 1:** Primers for qRT-PCR

Transcript	Primer sequences	Product length (bp)	Annealing temp(°C)	Accession Number
GPRC6A	F:CCGGGACATATCATAATTGGG R:CATTGCCACTGTGACTTCTGT	234	58	NM_148963
PCNA	F:CCTGCTGGGATATTAGCTCCA R:CAGCGGTAGGTGTCGAAGC	109	61	NM_002592
c-Fos	F:GAGGGAGCTGACTGATACAC R:CTCGGGGTAGGTGAAGACGA	568	60	NM_005252
MMP2	F:TACAGGATCATTGGCTACACACC R:GGTCACATCGCTCCAGACT	90	60	NM_004530
MMP9	F:TGTACCGCTATGGTTACACTCG R:GGCAGGGACAGTTGCTTCT	97	58	NM_004994
VEGF	F:CAAGACAAGAAAATCCCTGTGG R:CCTCGGCTTGTCACATCTG	161	58	M32977
BMP3	F:GCAGGGAGAGAGACCGAAG R:TGGACCGTGCTGTACCTGT	163	58	NM_001201
PSA	F:GTGTGTGGACCTCCATGTTATT R:CCACTCACCTTTCCCCTCAAG	160	60	AJ512346
RUNX2	F:TCAACGATCTGAGATTTGTGGG R:GGGGAGGATTTGTGAAGACGG	81	58	NM_001015051
OCN	F:GGCGCTACCTGTATCAATGG R:GTGGTCAGCCAACTCGTCA	110	60	NM_199173
HSD17B11	F:CTGGCGAAGTGCGTGAGATT R:GAGTACGCTTTCCCAATTCCAT	133	58	NM_016245
HSD3B1	F:CACATGGCCCGCTCCATAC R:GTGCCGCCGTTTTTCAGATTC	90	58	NM_000862
AKR1C3	F:TCTGGGATCTCAACGAGACAA R:TGGAACTCAAAAACCTGCACG	207	58	NM_003739
Cyclophilin A	F:CTGCACTGCCAAGACTGAGTG R:CCACAATGTTCATGCCTTCT	77	58	NM_008907

### Xenograft model

Athymic nude mice were purchased from Charles River laboratories. Mice were maintained and used in accordance with recommendations as described (National Research Council. 1985; Guide for the Care and Use of Laboratory Animals DHHS Publication NIH 86-23, Institute on Laboratory Animal Resources, Rockville, MD) and following guidelines established by the University of Tennessee Health Science Center Institutional Animal Care and Use Committee. The animal study protocol was approved by the institutional review board at University of Tennessee Health Science Center Institutional Animal Care and Use Committee.

GPRC6A edited PC-3 cells or control cells were labeled with a lentiviral vector expressing luciferase (pLenti-UBC-Luc2-T2A-mKate) and 2 × 10^6^ cells were mixed with Matrigel (Corning, #356234) and subcutaneously injected into the left and right flank of 5-week-old nude mice, and the mice were administrated 3 ng/g osteocalcin or vehicle per day by IP injection. Tumor growth was monitored over a 28-day period using a Xenogen imaging system (PerkinElmer). Mice were then sacrificed and tumors were dissected free of connective tissue before analysis. Immunohistochemistry (IHC) analysis was accomplished by Vectastain ABC kit (Vector Laboratories, PK-6200), and the antibodies used for IHC were obtained from commercial sources and diluted for use as follows: GPRC6A (1:100; ASSAY BIOTECHNOLOGY, # G321); and RUNX2 (1:100, Abcam, #b23981); PCNA (1:100, Invitrogen, #133900). Staining intensity was performed by assessing Vector NovaRed color (Vector Laboratories, SK-4800) in 3 sections of tumor for each antibody.

### Statistical analysis of data

We evaluated differences between groups by one-way analysis of variance, followed by *a post-hoc* Tukey's test. Significance was set at *p* < 0.05. All values are expressed as means ± SEM. All computations were performed using the Statgraphic statistical graphics system (STSC Inc.).

## Results

### Human GPRC6A transcripts are expressed in prostate cancer cells

**Fig. 1:**
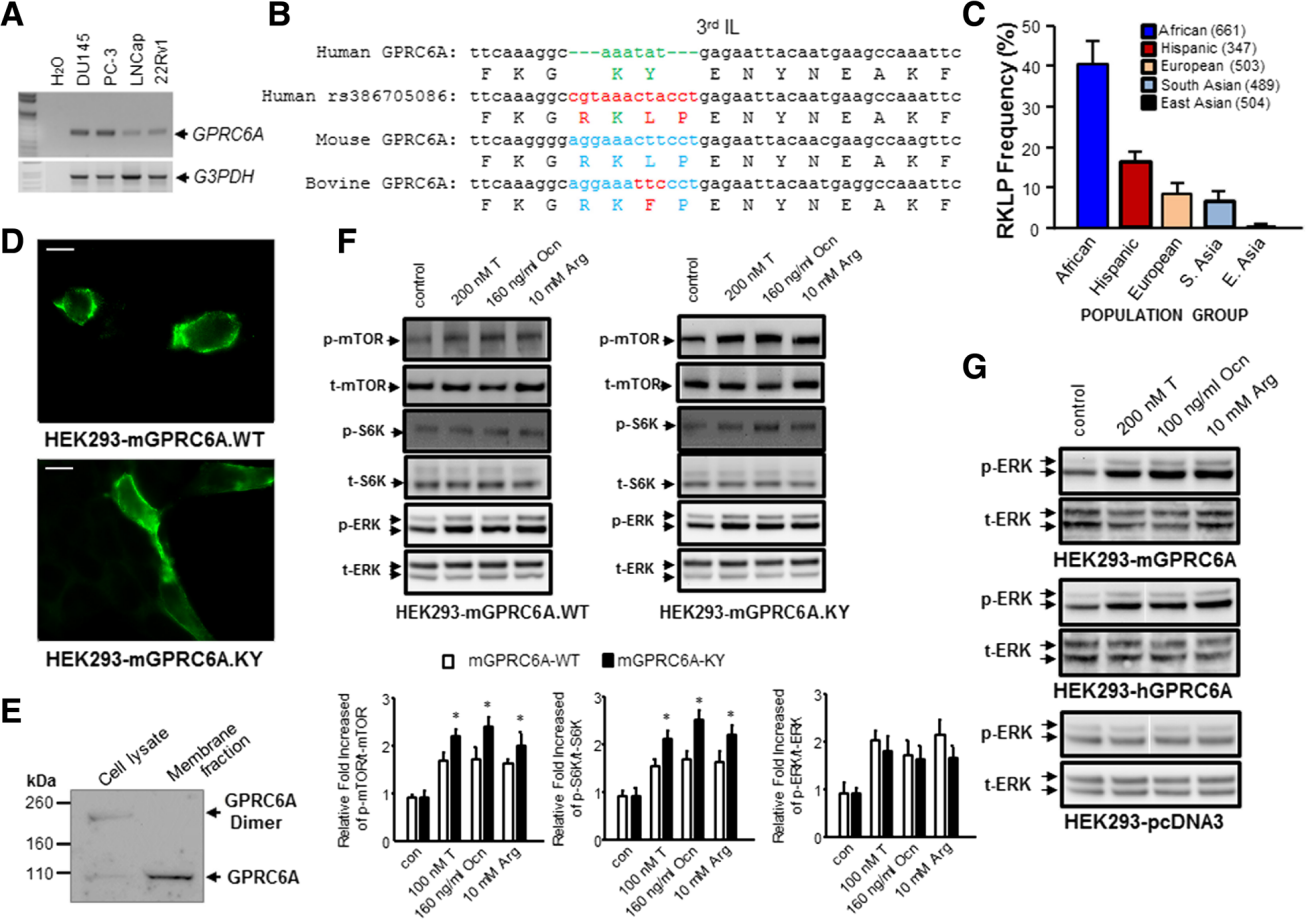
Expression and function of GPRC6A. **a** RT-PCR analysis of GPRC6A message expression in human prostate cancer cell lines, DU145, PC-3, LNCap and 22Rv1. **b** Comparison of nucleotides and amino acid sequences of GPRC6A 3rd IL from human GPRC6A reference (NM_148963) sequence showing the K..Y insertion/deletion, and the presence of the ancestral RKLP sequence in the human rs386705086 GPRC6A SNP, mouse GPRC6A (NM_153071) and bovine GPRC6A (XM_010808417) sequences. **c** Data from www.1000genomes.org showing distribution of the RKLP allele in human grouped by geographical area. Each population group contains a minimum of 4 and maximum of 7 subpopulations. The ancestral RKLP polymorphism is overrepresented in people of African descent. **d** Comparison of GPRC6A cell-surface distribution in HEK-293 cells transfected with mouse GPRC6A (*upper panel*) and “humanized” GPRC6A, where the “K..Y” sequences replaces the RKLP sequence in the mouse cDNA (*lower panel*). Transfected cells were fixed and stained with anti-myc antibodies and imaged on a Zeiss Axiolmager II microscope. Bars are 10 μm. **e** Immunoblot analysis of total and membrane-associated GPRC6A from PC-3 cells transfected with the mouse GPRC6A cDNA. Consistent with membrane expression in **d**, GPRC6A was enriched in the membrane fraction. **f** mGPRC6A-WT (*left panel*) and mGPRC6A-KY mutant (*right panel*) activity as assessed by phosphorylation of mTOR, p70S6K (S6K), and ERK. * Significant difference from control group and sgRNA3 group at *p* < 0.05 (n ≥ 4). **g** Comparison of mGPRC6A and hGPRC6A response to the ligands, 200 nM testosterone, 160 ng/ml osteocalcin and 10 mM L-Arg in HEK-293 cells transfected with mouse GPRC6A or human GPRC6A cDNAs

We confirmed that GPRC6A message is present in several human prostate cancer cell lines [12], including androgen insensitive PC-3 cells (derived from lumbar metastasis), DU145 cells (derived from CNS metastasis), and 22Rv1 cells (derived from androgen insensitive xenograft), and androgen sensitive LNCap cells (derived from lymph node metastasis) (Fig. 1a). The levels of GPRC6A transcripts were highest in PC-3 and DU145 cell lines.

Because of questions regarding the cell surface expression of GPRC6A [28, 29], and the importance of the 3rd intracellular loop (3rd IL) in GPCR membrane trafficking [39], we sequenced this region in DU145, PC-3, LNCap and 22Rv1 human prostate cancer cell lines [28]. The cDNA sequence encoding the 3rd IL from all four human prostate cancer cells had the AAATAT nucleotide sequence encoding the “K..Y” amino acid sequence [the human reference at amino acid 774 (Lys774Tyr775)] shown in Fig. 1b. The human GPRC6A 774-KY-775 motif in the 3rd IL represents an insertion/deletion that replaced the ancestral 774-RKLP-777 sequence [variation rs386705086 (p.Lys_Tyr775 replace to ArgLysLeuPro); except for 775-RK**F**P-778 in the bovine sequence] (Fig. 1b). The “K..Y” deletion/insertion emergenced only in hominids, and is found in greater than 84% of humans [38, 40]. The ancestral “RKLP” sequence present in all other animal species is considered to be a polymorphism (rs386705086) in humans (Fig. 1b), and is retained with an allele frequency 0.6% in people of East Asian descent, 6.6% of South Asians, 8.2% of Europeans, 16.6% of Hispanics, and 40.1% in people of African descent (Fig. 1c, Data from www.1000genomes.org). The emergence of the “K..Y” polymorphism only in hominids and its preservation during evolution suggests a selection pressure that modified the functions of this allele in the majority of humans [33].

### Cell distribution and ligand activation of GPRC6A

Next, we compared the cell distribution and function of a wild-type mouse GPRC6A cDNA constructs (mGPRC6A.WT) and a mutated mouse GPRC6A construct in which the “RKLP” sequence was replaced with the human “K..Y” sequence (mGPRC6A.KY). Results from transfection of HEK-293 cells with the mGPRC6A.WT and mGPRC6A.KY are shown in Fig. 1d. We observed no significant differences in the cellular distribution of mouse GPRC6A containing the RKLP sequence and the mouse GPRC6A cDNA containing the K..Y sequence in 3rd IL. Both wild-type and “humanized” mouse GPRC6A were predominantly expressed on the surface of cells (Fig. 1d, upper and bottom panel, respectively). We also have compared the protein levels of GPRC6A from a total cell lysate and membrane fraction prepared from PC-3 cells (Fig. 1e). The western blot indicates that GPRC6A is enriched in the membrane fraction prepared from PC-3 cells (Fig. 1e).

To determine if the K..Y polymorphism affects ligand-dependent activation of GPRC6A in these cells, we tested the ability of the GPRC6A ligands, L-Arg, osteocalcin and testosterone, to activate ERK-signaling. Transfection of the wild-type or “humanized” mouse GPRC6A into HEK-293 cells resulted in similar ERK signaling in response to ligand stimulation of GPRC6A (Fig. 1f). HEK-293 cells transfected with the human or mouse wild-type GPRC6A cDNAs also equally increased ERK activity in response to 10 mM L-Arg, 160 ng/ml osteocalcin, or 200 nM testosterone, respectively (Fig. 1g). However, the K..Y mutant preferentially activated mTOR and p70S6K phosphorylation in the mTOR pathway compared to the wild-type RKLP mouse GPRC6A (Fig. 1f).

### Editing GPRC6A in PC-3 cells using CRISPR/Cas9

**Fig. 2:**
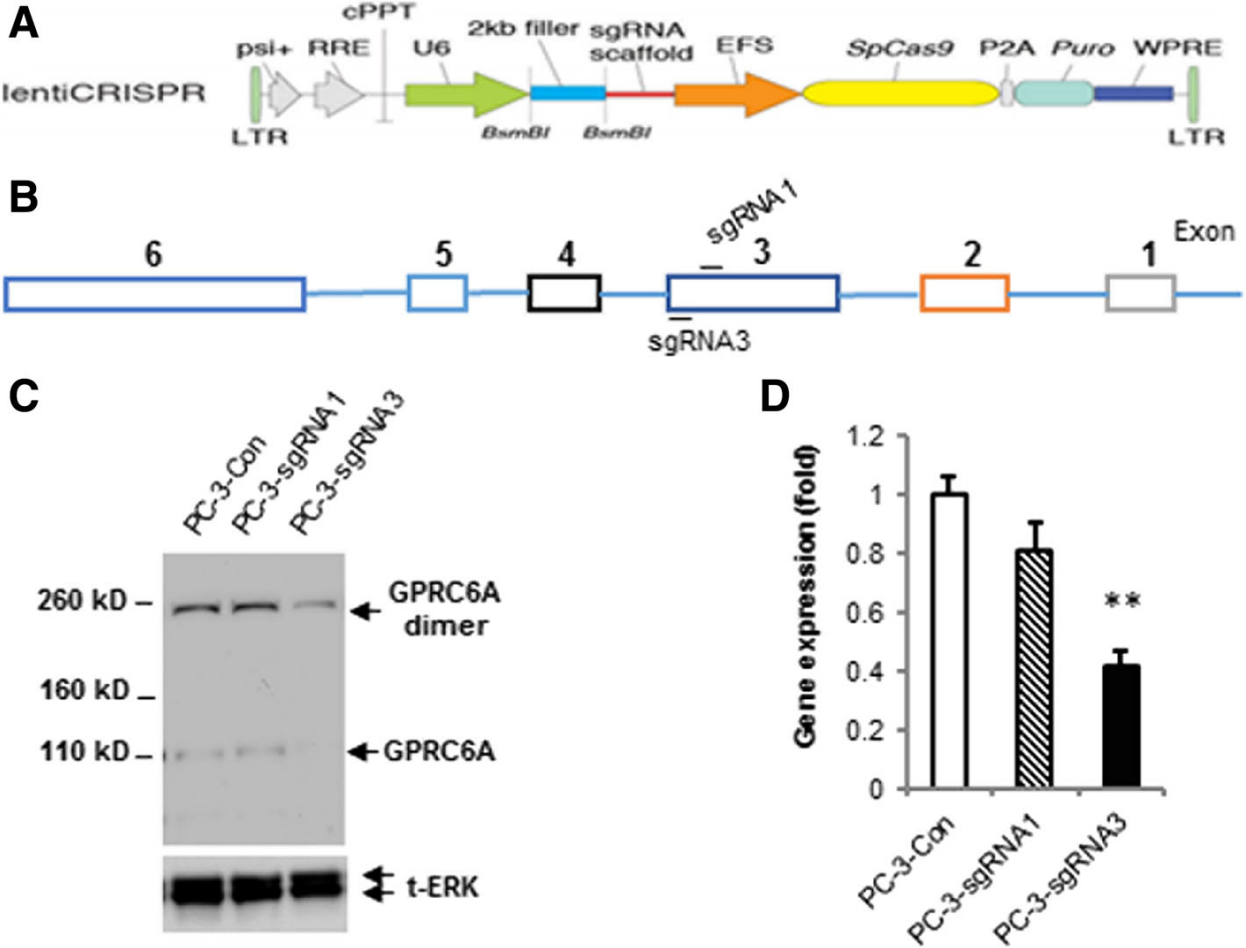
Editing GPRC6A in PC-3 cells using CRISPR/Cas9. Strategy of targeting GPRC6A in human prostate cancer PC-3 cells using CRISPR/Cas9 and the vector of lentiCRISPR v2 (the figure is adopted from http://genome-engineering.org/gecko/wp-content/uploads/2013/12/lentiCRISPRv2-and-lentiGuide-oligo-cloning-protocol.pdf) (**a**) and GPRC6A gene structure (**b**). *Blue bars* show sgRNA1 and sgRNA3 location in exon 3 of GPRC6A gene. Short guide RNAs (sgRNA) designed to target exon 3 of GPRC6A gene. The controls were empty vector, with no inserts in this region. CRISPR/Cas9 editing in PC-3 human prostate cancer cells decreased GPRC6A protein by Western blot (**c**) and mRNA by real-time PCR (**d**). ** Significant difference from control group and sgRNA3 group at *p* < 0.01 (*n* ≥ 4)

To further investigate the function of endogenous human GPRC6A, we used CRISPR/Cas9 editing to mutate the GPRC6A gene in the PC-3 s cells. We used a lentiCRISPR vector (Fig. 2a) and designed sgRNAs (sgRNA1 and 3) against exon 3 of GPRC6A (Fig. 2b). We designed two sgRNAs against exon 3 to avoid non-specific effects of the CRISPR/Cas9 system. Genomic DNA PCR products were cloned into a plasmid and the GPRC6A gene targeted by the sgRNAs was sequenced to analyze the targeted GPRC6A genomic region in the transfected PC-3 cells. This analysis revealed that the sequence of the GPRC6A gene was specifically altered in ~ 80% of the cells that would disrupt gene function, including insertions or deletions at the sites targeted by sgRNA1 and 3 (Additional file 1: Figure S1). Western blotting (Fig. 2c) and real-time PCR (Fig. 2d) confirmed that GPRC6A protein and mRNA expression were significantly decreased in PC-3 cells edited using sgRNA-1 and sgRNA3. Furthermore, editing PC cells with sgRNA3 resulted in a greater decrease in GPRC6a protein and mRNA expression than that observed with sgRNA1s (Fig. 2c and d).

### CRISPR/Cas9 editing of GPRC6A in PC-3 cells attenuates ligand dependent prostate cancer responses in vitro

**Fig. 3:**
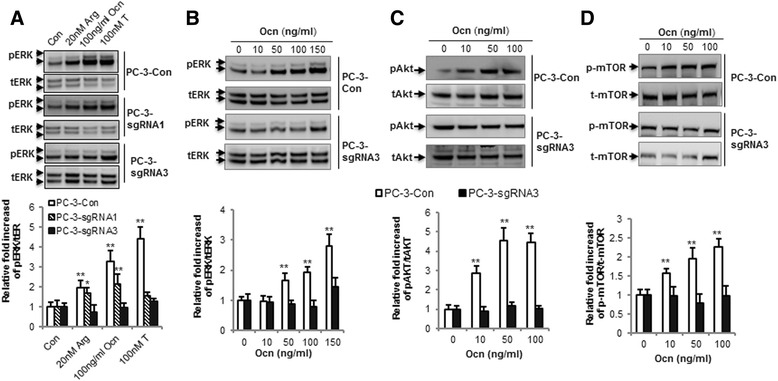
Editing GPRC6A attenuated osteocalcin-induced intracellular signaling activation in PC-3 cells. **a** Comparison of Arg, osteocalcin and testosterone activated ERK phosphorylation in PC-3 control, PC-3-sgRNA1, PC-3-sgRNA3 cells. Dose dependent effect of osteocalcin on ERK phosphorylation (**b**), AKT phosphorylation (**c**), and mTOR phosphorylation (**d**) was blocked in PC-3 cells with CRISPR/Cas9 editing of the GPRC6A gene locus. Protein intensity in the Western blot was analyzed by Image J. * and ** represent significant differences from control group and stimulated groups at *p* < 0.05 and *p* < 0.01 (*n* ≥ 4)

In prior studies we show that ligand-mediated activation of GPRC6A is coupled to ERK and AKT phosphorylation [37, 41]. Therefore, we assessed ERK activation in response to GPRC6A ligands in PC-3-sgRNA1, PC-3-sgRNA3, and PC-3 control cells. We found that 20 mM L-Arg, 100 ng/ml osteocalcin and 100 nM testosterone stimulated phospho-ERK activity in control PC-3 cells (Fig. 3a, upper panel), and this response was significantly decreased in PC-3 cells edited using sgRNA1 and sgRNA3 guide RNAs (Fig. 3a). In PC-3-sgRNA3 cells there was a significant reduction in ERK activity stimulated by L-Arg, osteocalcin and testosterone (Fig. 3a, lower panel), whereas PC-3-sgRNA1 cells showed a partial response to L-Arg and osteocalcin, but no response to testosterone at the concentration tested (Fig. 3a, lower panel).

To test the dose-dependent effects of ligand activation of GPRC6A, we examined the effects of osteocalcin at doses of 0, 10, 50, 100, 150 ng/ml on ERK and Akt phosphorylation in control and PC-3-sgRNA3 cells. We observed that Osteocalcin stimulated ERK (Fig. 3b) and AKT (Fig. 3c) phosphorylation in a dose-dependent manner in control PC-3 cells transfected with empty lentiCRISPR vector. Osteocalcin stimulated ERK and AKT phosphorylation were significantly attenuated in PC-3-sgRNA3 cells (Fig. 3b and c). Osteocalcin also stimulated the phosphorylation of mTOR, a downstream effector of ERK and AKT, in control PC-3 cells, but mTOR phosphorylation was significantly reduced in osteocalcin stimulated PC-3-sgRNA3 cells (Fig. 3d).

**Fig. 4:**
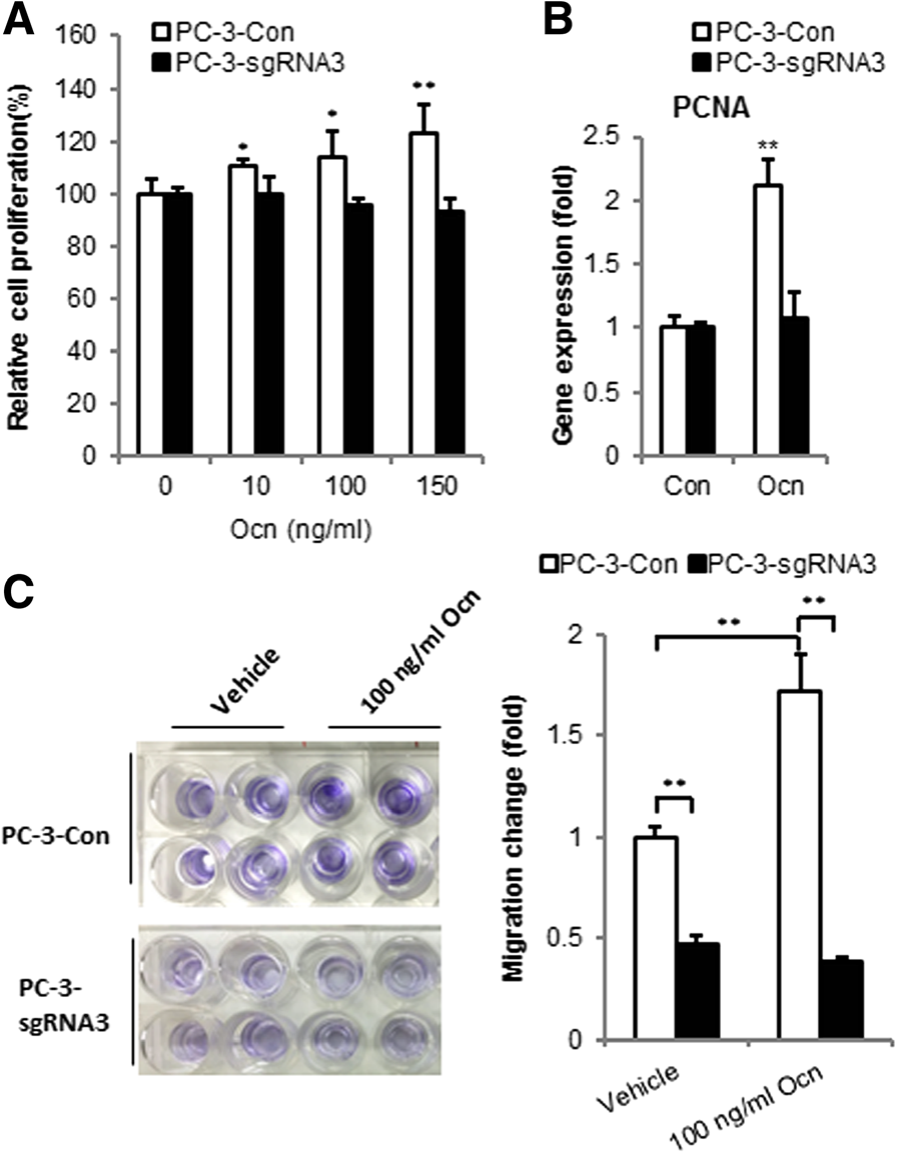
CRISPR/Cas9 editing GPRC6A in PC-3 cells decreased cell proliferation and migration in response to osteocalcin. **a** Dose-dependent effect of osteocalcin on PC-3 cell proliferation. Cell proliferation was examined days after seeding by using MTT method (*n* = 8). **b** Effect of osteocalcin on cell proliferation marker PCNA gene expression. Cells were analyzed at 24 h after seeding by using MTT method (*n* = 8). **c** Osteocalcin significantly promoted PC-3 control cells migration and its effect was significantly blocked by CRISPR/Cas9 editing of GPRC6A in PC-3-sgRNA3 cells. Cells were examined by using CytoSelect 24-well cell migration Colorimetric Format assay kit at 24 h after 100 ng/ml osteocalcin-treated. * and ** indicate significant differences from control group and stimulated groups at *p* < 0.05 and *p* < 0.01 (*n* = 4)

We assessed the effects of GPRC6A on prostate cancer growth and cell migration. The reduced expression of GPRC6A in PC-3-sgRNA3 cells had a small but significant effect on osteocalcin-stimulated increase in PC-3 cell number (Fig. 4a). GPRC6A gene editing of exon 3 using CRISPR/Cas9 significantly decreased osteocalcin-induced PCNA expression, a cell proliferation marker gene (Fig. 4b). Cell migration was also significantly decreased in PC-3-sgRNA3 cells compared to PC-3 controls in the absence of osteocalcin; and the addition of osteocalcin to the culture media resulted in a significant 72% increase in control PC-3 cell migration, and this response was completely inhibited in PC-3-sgRNA3 cells (Fig. 4c).

**Fig. 5:**
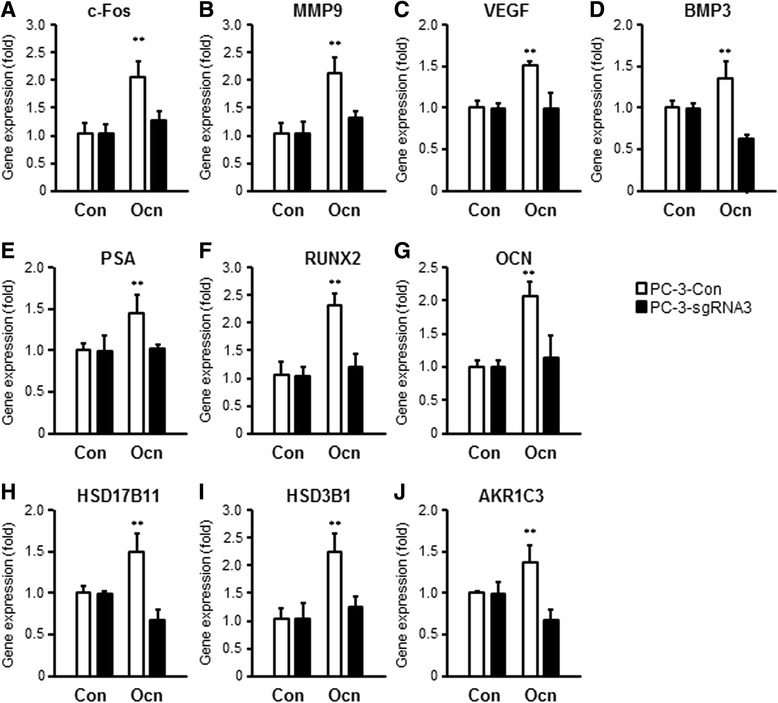
CRISPR/Cas9 editing of GPRC6A impairs osteocalcin-induced gene expression. Control and CRISPR/Cas9 edited PC-3 cells were treated with 100 ng/ml osteocalcin or vehicle for 24 h, gene expression was analyzed by qRT-PCR. c-Fos gene expression (**a**); cell migration markers, MMP9 (**b**), VEGF (**c**) and BMP3 gene expression (**d**); prostate cancer progression markers, PSA (**e**), RUNX2 (**f**), and OCN gene (**g**) expression. Testosterone biosynthesis pathway enzymes, HSD17B11 (**h**) HSD3B1 (**i**), and AKR1C3 gene expression (**j**). ** indicates a significant difference from control and stimulated groups at *p* < 0.01 (*n* = 6)

We also examined by real-time PCR the effects of ligand stimulated GPRC6A on a panel of genes known to be associated with prostate cancer progression. We found that the mRNA levels of *c-Fos* and the migration-related genes, MMP9, VEGF and BMP3, were significantly increased by osteocalcin stimulation of PC-3 control cells, but not in PC-3-sgRNA3 cells (Fig. 5a–d). Prostate specific antigen [42] and Runt-related transcription factor 2 (RUNX2), a bone-specific transcriptional regulator expressed in metastatic prostate cancer cells, are regulated by ligand activation of GPRC6A [37]. We observed that osteocalcin significantly stimulated *PSA*, *RUNX2* and osteocalcin (*OCN*) message levels in PC-3 control cells, but not in PC-3-sgRNA3 cells (Fig. 5e–g). We also tested the effects of GPRC6A activation on testosterone biosynthesis in prostate cancer cells [15]. Osteocalcin addition to PC-3 control cells significantly increased *HSD17b11, HSD3B1*, and *AKR1C3*, which are involved in testosterone biosynthesis. In contrast, osteocalcin did not increase the expression of these genes in PC-3-sgRNA3 cells (Fig. 5h–j).

### CRISPR/Cas9 editing of GPRC6A in PC-3 cells decreases prostate cancer tumor growth in vivo

**Fig. 6:**
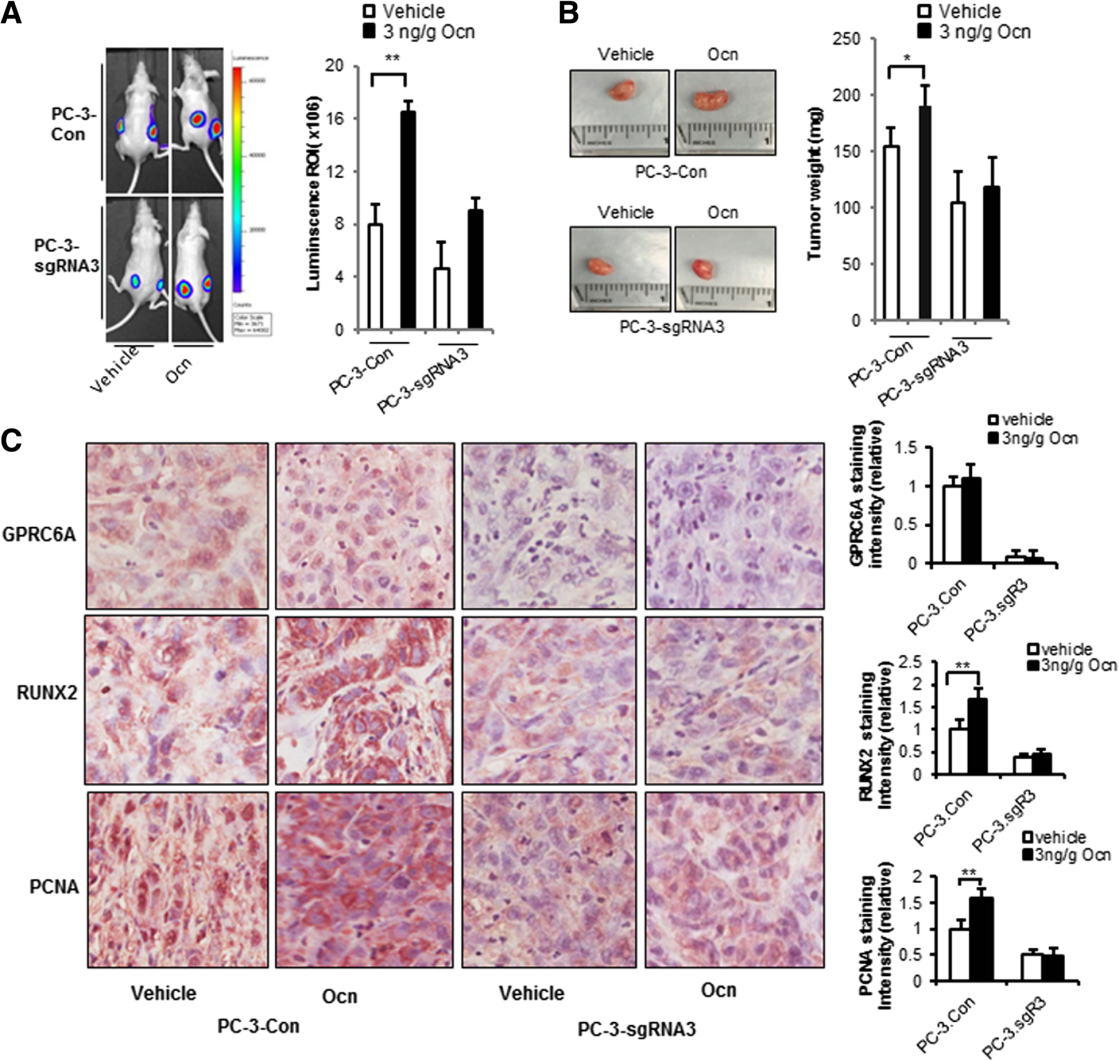
CRISPR/Cas9 editing of GPRC6A decreased prostate cancer tumor growth in xenograft models. Tumor development was monitored over a 28-day period by measuring whole-body luciferase activity. Mice were then sacrificed at day 29. **a** Editing GPRC6A in PC-3 cells inhibits growth of tumor xenograft in nude mice. PC-3 cells xenograft were detected by in vivo bioluminescence imaging. BLI ratio of intensity (ROI) was shown in right bar graft. **b** Gross appearance and tumor weight of each group. **c** GPRC6A, RUNX2 and PCNA protein expression in tumor tissues, analyzed by immunohistochemistry. *Bar graphs* on the right quantify GPRC6A, RUNX2, and PCNA expression by staining intensity after specific antibody, secondary antibody and histochemical color development. ** Significant difference from control group and stimulated group at *p* < 0.01 (*n* = 3)

Finally, to investigate the effects of GPRC6A on regulation of prostate cancer cell progression in vivo, we performed human xenograft studies in nude mice. Human PC-3 control cells and PC-3-sgRNA3 cells expressing a luciferase reporter were injected subcutaneously (s.c.) into the lower flank of 5 week-old nude mice followed by treatment with either vehicle or osteocalcin at a daily dose of 3 ng/g by IP injection. Tumor growth was assessed by luciferase intensity (Fig. 6a) and mass was assessed by weighing dissected tumors (Fig. 6b). In vehicle treated mice, implanted control PC-3 cells exhibited significant increases in tumor growth (Fig. 6a) and weight (Fig. 6b) compared to mice implanted with GPRC6A PC-3-sgRNA3 cells. Osteocalcin treatment significantly increased tumor growth and weight in PC-3 control xenografts (Fig. 6a and b). In contrast, osteocalcin administration resulted in an attenuated, but non-significant increase in tumor growth and no change in tumor gross size or weight in mice implanted with PC-3-sgRNA3 cells (Fig. 6a and b). Immunohistochemical staining revealed a dramatic reduction in the protein expression of GPRC6A in the tumors of PC-3-sgRNA3 cells xenografts compared to PC-3 control cells (Fig. 5c, upper panel). Osteocalcin-stimulation did not alter the expression of GPRC6A (Fig. 5c, upper panel). We observed a significant increase in PCNA and RUNX2 protein levels in tumors derived from PC-3 control cells treated with osteocalcin (3 ng/g Osteocalcin, IP daily for 5 weeks); in contrast, treatment of PC-3-sgRNA3 xenografts with osteocalcin did not result in increased levels of PCNA and RUNX2 (Fig. 6c, middle and lower panels, respectively).

## Discussion

In this study we tested if human GPRC6A has important tumor promoting effects in prostate cancer. Using a panel of human prostate cancer cell lines, we confirmed that human GPRC6A transcripts are expressed in all prostate cancer cell lines tested. Because cell surface membrane localization of human GPRC6A has been questioned [28, 29, 33, 43], we sequenced the 3rd intracellular loop, a region of GPCRs that binds β-arrestins and regulates membrane trafficking [28, 29]. We identified the presence of only the “K..Y” sequence in the 3rd IL in human GPRC6A. This sequence is noteworthy, because it evolved only in hominids and replaces the ancestral “RKLP” sequence present in all other species [40]. Comparision of the cellular localization of GPRC6A in HEK-293 cells transfected with wild-type mouse and a humanized mouse GPRC6A cDNA with the “K..Y” sequence showed that both localized to the cell surface. Human GPRC6A containing the K..Y sequence (human reference sequence) has been shown by others to localize to the cell curface when expressed in HEK-239 T cells [15].

The cell surface localization of the ‘humanized’ mouse GPRC6A, however, differs from other studies of the human GPRC6A that show a punctate, intracellular-like distribution of this receptor in transfected HEK-293 cells [33, 44]. Lv et al. [43] found that human GPRC6A exhibited approximately 30% cell surface expression, whereas the closely related calcium sensing receptor, CasR, which has the RKLP sequence in its 3rd IL, exhibited >90% cell surface expression. Our data also, differ from the analysis of human/mouse chimeric GPRC6A constructs that replaced the entire 3rd IL, and concluded the respective K..Y and the RKLP inserts in human and mice determined the intracellular and cell surface membrane expression of these receptors. Replacing the RKLP sequence, however, with K..Y in the mouse receptor is not sufficient for intracellular retention of the mouse GPRC6A and that additional sequences in the 3rd IL or other domains likely regulate cell surface expression of GPRC6A [15, 33, 44].

Nevertheless, in heterologous cell systems transfected with different mouse and human GPRC6A constructs, we found that ligands activated GPRC6A encoded by both mouse and human constructs, irrespective of whether they contained the “K..Y” or “RKLP” polymorphisms. Indeed, transfection in HEK-293 cells of cDNAs encoding the mouse GPRC6A with the “RKLP” sequence, the human GPRC6A with the “K..Y” sequence, and a “humanized” mouse GPRC6A created by replacing the RKLP with “K..Y” sequence, all imparted the ability of ligands to stimulate ERK, Akt and mTOR phosphorylation.

Osteocalcin activation of GPRC6A has previously been shown to stimulate mTOR phosphorylation in myocytes and fibroblasts [10, 41]. mTOR can be activated by cell surface receptors through PI3K/Akt or through an evolutionarily conserved intracellular amino acid lysosomal mTORC1 signaling pathway involving RAG GTPases, Ragulator, and vaculoar H^+^-ATPase [45]. Interestingly, the presence of the “K..Y” polymorphism resulted in a greater mTOR response, suggesting that the “K..Y” polymorphism may alter signaling through mTOR. There is a precident for β-arrestin-dependent regulation of endosomal signaling of the PTH GPCR [39], and it is tempting to speculate that the “K..Y” polymorphism in human GPRC6A may have evolved to alter GPRC6A’s interactions with β-arrestin [16], and the kinetics of receptor recycling to allow for participation in this intracellular amino acid sensing pathway [45]. Further studies are needed to test this possibility.

More importantly, we established that endogenous human GPRC6A functionally responds in both in vitro and in vivo model systems. We used CRISPR/Cas9 to delete endogenous GPRC6A in androgen insensitive human PC-3 cells. GPRC6A edited PC-3 cells exhibited an attenuated response to L-Arg, osteocalcin and testosterone stimulation of ERK, Akt, and mTOR phosphorylation compared to controls, pathways involved in advanced prostate cancer and emergence of hormone-refractory disease [46]. In addition, activation of GPRC6A stimulated prostate cancer cell line proliferation and migration, whereas editing GPRC6A in PC-3 cells reduced cell proliferation and transcription of *PCNA* and *c-Fos*. CRISPR/Cas9 editing of PC-3 cells showed impaired ligand-stimulated expression of *MMP9, VEGF, BMP3, PSA, RUNX2*, and *OCN*, which are involved in prostate cancer progression [2, 20, 47, 48]. In addition, GPRC6A edited PC-3 cells exhibited reduced ligand stimulated expression of transcripts encoding key enzymes regulating intra-tumor androgen biosynthesis, including 17-beta-hydroxysteroid dehydrogenase 11 (HSD17B11), hydroxy-delta-5-steroid dehydrogenase, 3 beta- and steroid delta-isomerase 1 (HSD3B1), and aldo-keto reductase family 1, member C3 (ARK1C3). Since PC-3 cells are androgen-independent with no or weak androgen receptor (AR) activity [49, 50], GPRC6A may respond to intra-prostatic androgen synthesis and contribute to the high metastatic potential of PC-3 cells. Osteocalcin activation of GPRC6A may indirectly promote androgen synthesis through stimulation of IL-6 [10], a cytokine that can promote androgen synthesis in prostate cancer cells through enhancing AKR1C3 transcription [51]. Overall, these findings are consistent with prior in vitro studies showing that activation of GPRC6A in human PC-3 and 22Rv1 cells results in ERK phosphorylation, cell proliferation, and chemotaxis [12]; that knockdown of GPRC6A by siRNA inhibited PC-3 prostate cancer cell migration and invasion, and that overexpression of GPRC6A promoted prostate cancer epithelial-mesenchymal transition [20].

Finally, the mouse PC-3 xenografts showed that activation of GPRC6A with osteocalcin enhances primary tumor growth, and that CRISPR/Cas9 induced mutations in GPRC6A in PC-3 cells resulted in reduced primary tumor growth and the further enhancement of tumor growth in response to osteocalcin administration. PCNA and RUNX2 expression was decreased and the ability of osteocalcin to increase PCNA and RUNX2 expression was attenuated in xenografts derived from PC-3 cells in which GPRC6a levels were reduced. These data, along with the observations that GPRC6A is upregulated in human prostate cell lines [12], and GWAS studies that have identified other polymorphisms in the GPRC6A gene associated with prostate cancer progression in men of Asian descent [21–23], support the notion that GPRC6A has biological functions in prostate cancer.

## Conclusion

Available evidence suggests that GPRC6A and its ligands modulate the progression of prostate cancer. We previously demonstrated that GPRC6A can directly promote prostate cancer cell proliferation, migration, and in vivo tumor growth [12]. The current study shows that by activating GPRC6A, signaling via ERK, AKT, and mTOR is increased in prostate cancer cells. In addition, activation of GPRC6A can stimulate androgen biosynthesis pathways to regulate intra-tumoral steroidogenesis in prostate cancer cells [52], and in testicular Leydig cells to stimulate circulating testosterone concentrations. Our findings raise the possibility that the presence of osteocalcin in the bone micro-environment potentially creates paracrine signaling pathways that might explain the propensity of CRPC to metastasize to bone [53]; and expression of osteocalcin in malignant prostate cell lines and prostate cancer specimens potentially leads to autocrine activation of GPRC6A [54]. Our findings also suggest that GPRC6A, by mediating the non-genomic effects of testosterone [55], may provide alternative pathways to androgen receptor signaling in prostate cancer [26]. Finally, the persistence of the ancestral RKLP sequence and its predominant expression in humans of African descent suggest that this human polymorphism may contribute to the racial disparities in diseases associated with GPRC6A, including Metabolic Syndrome [56] and prostate cancer [57]. As such, GPRC6A is “a target of interest” for treating prostate cancer and warrants further efforts to develop antagonists for this receptor as potential treatments for prostate cancer.

## Additional file

Additional file 1:
**Figure S1.** Targeting GPRC6A gene in PC-3 cells using CRIPR/Cas9 system. (DOCX 89 kb)

## Abbreviations

Cas9: CRISPR-associated protein 9 nuclease
CRISPR: Clustered regularly interspaced short palindromic repeats
ERK: Extracellular signal-regulated kinases
GPRC6A: G Protein-Coupled Receptor Class C Group 6 Member A
K..Y: The human reference at amino acid 774 (Lys774Tyr775)
MetS: Metabolic syndrome
RKLP: Variation rs386705086 (p.Lys_Tyr775 replace to ArgLysLeuPro)
sgRNA: Short guide RNA
