# Supplementary material for: CRISPR/Cas9 targeting of GPRC6A suppresses prostate cancer tumorigenesis in a human xenograft model
Source: J Exp Clin Cancer Res. 2017 Jun 28;36:90. doi: 10.1186/s13046-017-0561-x (PMC5490090; doi:10.1186/s13046-017-0561-x)
Supplement: Additional file 1: — Figure S1. Targeting GPRC6A gene in PC-3 cells using CRIPR/Cas9 system. (DOCX 89 kb) [file 13046_2017_561_MOESM1_ESM.docx]

**CRISPR/Cas9 targeting of GPRC6A suppresses prostate cancer tumorigenesis in a human xenograft model**

**Ruisong Ye^1^, Min Pi^1^, John V Cox^2^, Satoru K Nishimoto^2^, L Darryl Quarles^1^**

Department of ^1^Medicine and ^2^Microbiology, Immunology and Biochemistry, University of Tennessee Health Science Center, 19 S Manassas St. Memphis, TN 38163

**
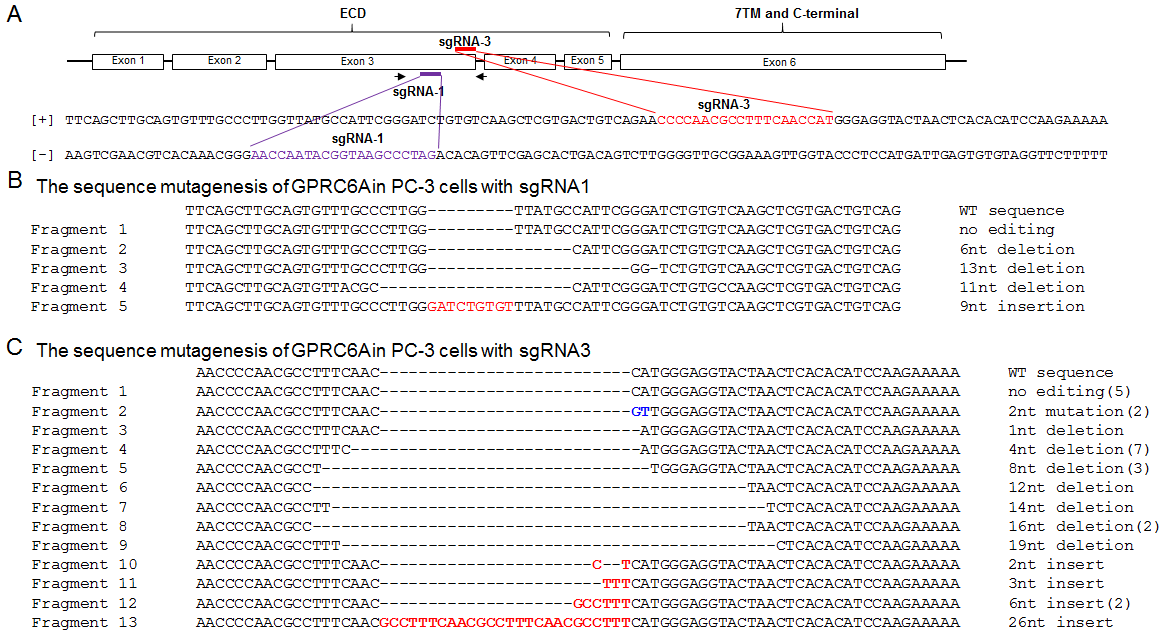
**

**Supplemental Figure 1. Targeting GPRC6A gene in PC-3 cells using CRIPR/Cas9 system.** (A) The structure of GPRC6A gene, and location and sequences of GPRC6A.sgRNA1 (purple short bar) and GPRC6A.sgRNA3 (red short bar). (B and C) The sequence of GPRC6A in PC-3 cells targeted with GPRC6A.sgRNA1 (B) and GPRC6A.sgRNA3 (C). PCR products from PC-3 cells targeted by GPRC6A.sgRNA1 and GPRC6A.sgRNA3 were cloned and sequenced. In DNA isolated from PC-3 cells with the GPRC6A.sgRNA1 mutation, we cloned and sequenced 5 PCR fragments. We found 4 out of 5 clones had either insertions or deletions in targeted region that would disrupt gene function (Supplementary Figure 1B). In GPRC6A.sgRNA3 mutation, we sequenced a total 28 PCR product clones. We found 23 out of 28 clones contained mutations consisting of either insertions or deletions in targeted region (Supplementary Figure 1C). The blue letters indicate changed nucleotides. The red letters indicate nucleotide insertions. The number in parentheses indicates the number of times a particular sequence was observed. The location of the PCR primers used to amplify this region of the GPRC6A gene is indicated by arrows corresponding to hGPRC6A.Fmut and hGPRC6A.Rmut as described in methods.
